# Combination of High-Density and Coherent Mapping for Ablation of Ventricular Arrhythmia in Patients with Structural Heart Disease

**DOI:** 10.3390/jcm11092418

**Published:** 2022-04-26

**Authors:** Vanessa Sciacca, Thomas Fink, Leonard Bergau, Guram Imnadze, Mustapha El Hamriti, Denise Guckel, Martin Braun, Moneeb Khalaph, Philipp Sommer, Christian Sohns

**Affiliations:** Clinic for Electrophysiology, Herz- und Diabeteszentrum NRW, Ruhr-Universität Bochum, 32545 Bad Oeynhausen, Germany; vsciacca@hdz-nrw.de (V.S.); tfink@hdz-nrw.de (T.F.); lbergau@hdz-nrw.de (L.B.); gimnadze@hdz-nrw.de (G.I.); melhamriti@hdz-nrw.de (M.E.H.); dguckel@hdz-nrw.de (D.G.); mbraun@hdz-nrw.de (M.B.); mkhalaph@hdz-nrw.de (M.K.); psommer@hdz-nrw.de (P.S.)

**Keywords:** VT ablation, coherent mapping, VT burden

## Abstract

The present study describes our experience with a new mapping approach for ventricular arrhythmia (VA) ablation in patients with structural heart disease (SHD). Consecutive patients undergoing catheter ablation for recurrent VA were analyzed. High-density mapping was conducted in all patients. In patients with inducible VA, local activation time (LAT) mapping and a novel vector-based mapping algorithm were implemented to analyze arrhythmia propagation. In case of focal tachycardia, the location of earliest activation was targeted. In VAs with re-entrant mechanisms, zones of slow conduction based on coherent mapping were ablated. Substrate modification was performed when pathologic electrograms were identified. Seventy-four patients were included. Sixty-five patients (87.8%) were male. Ischemic cardiomyopathy was the underlying disease in 35 patients (47.3%) and nonischemic cardiomyopathy was the underlying disease in 39 patients (52.7%). Mean left ventricular ejection fraction was 33.8 ± 9.9%. Non-inducibility of any VA was achieved in 70 patients (94.6%). Termination of VA was achieved in 93.5% of patients with stable VA. In 4 patients (5.4%), partial success was achieved. VA (*p* < 0.001), ATP (*p* < 0.001) and shock burden (*p* = 0.001) were significantly reduced after ablation. Mean arrhythmia-free survival after 12 months was 85.1 ± 4.7%. High-density mapping in combination with coherent mapping may facilitate the understanding of the tachycardia mechanism, providing targets for effective ablation.

## 1. Introduction

Ventricular arrhythmia (VA) is a leading cause of mortality and morbidity in patients with structural heart disease (SHD) [1,2]. Implantable cardioverter defibrillators (ICD) are the mainstay of therapy in patients with SHD in terms of primary or secondary prevention of VA and sudden cardiac death [3]. However, recurrent VA and subsequent ICD therapy can lead to repeat hospitalization, impaired quality of life and increased mortality [4,5,6]. Over the past decades, catheter ablation has emerged as an important treatment modality in patients with SHD and recurrent ventricular tachycardia (VT). Catheter ablation of VT has been shown to be associated with decreased frequencies of ICD shocks, VT storm and hospitalization [7]. An association of VA ablation and improved survival has been observed in a prospective multicenter registry study [8]. Nevertheless, catheter ablation was associated with relatively high rates of arrhythmia recurrence due to limited operator experience, limited understanding of tachycardia mechanisms and unclear procedural endpoints [9,10,11,12]. Over the past decade, overwhelming technological progress in terms of intracardiac mapping and catheter ablation has evolved. Multielectrode mapping catheters allowing for intracardiac ultrahigh-density mapping and steerable sheaths enabling visualization by electroanatomic mapping systems were developed. Recently, a novel module of an established electroanatomic mapping system has become available enabling coherent mapping based on conduction velocity vector analysis. This novel coherent mapping module has already been shown to be beneficial in patients with scar-related atrial tachycardia in terms of recognition of slow conduction areas and critical isthmus sites for defining ablation targets [13,14]. In the present study, we describe our institutional experience with this new mapping technology for VT ablation in patients with ischemic (ICM) or nonischemic cardiomyopathy (NICM).

## 2. Methods

### 2.1. Study Population

Data from consecutive patients undergoing radiofrequency-based catheter ablation due to recurrent VT between January 2020 and December 2021 at our institution were analyzed. All patients gave written informed consent before participating. The study followed the principals outlined in the Declaration of Helsinki and was approved by the local ethics committee (2019-563). The study was conducted as a prospective observational study as part of the institutional catheter ablation registry. Baseline, procedural and follow-up data were collected and statistically analyzed.

### 2.2. Peri- and Intraprocedural Management

Left ventricular thrombi were ruled out by transthoracic echocardiography in all patients prior to ablation. Atrial thrombus formation was ruled out by preprocedural transesophageal echocardiography in patients with high risk for left atrial or left atrial appendage thrombi. Interrogation of cardiac devices was conducted prior to ablation and tachycardia therapy was deactivated. Patients with medication of vitamin K antagonists received uninterrupted anticoagulation with a target INR of 2.0 to 3.0. In patients with preexisting treatment of direct oral anticoagulants (DOAC), uptake was paused one half-life before the procedure and continued within 4 h after ablation. All procedures were conducted in general anesthesia. During ablation continuous monitoring of heart rate, oxygen pressure, invasive measurement of blood pressure and body temperature was performed. Heparin administration for systemic anticoagulation was started after left heart access targeting an ACT > 300 s.

### 2.3. Ablation Protocol

**Figure 1:**
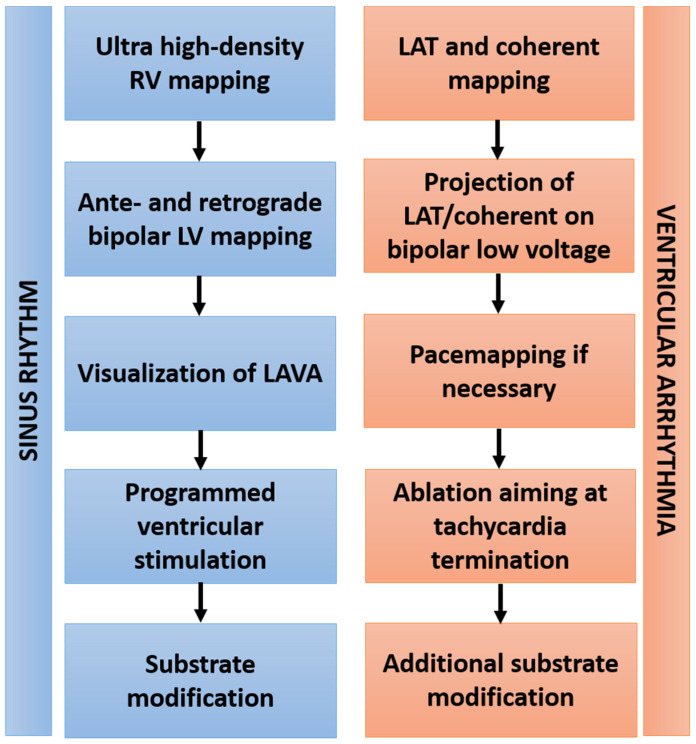
Workflow of VA ablation. Abbreviations: RV—right ventricle; LV—left ventricle; LAVA—local abnormal ventricular activation; LAT—local activation time.

Transseptal puncture was performed using a BRK-1 needle and a steerable sheath with electrodes allowing its visualization inside the 3D mapping system (8.5 Fr, ViziGo, large curve, Biosense Webster, Inc., Diamond Bar, Irvine, CA, USA). Two diagnostic catheters were introduced and positioned into the coronary sinus (6 Fr, Webster, Biosense Webster, Inc., Diamond Bar, Irvine, CA, USA) and the right ventricle (5 Fr, Webster, Biosense Webster, Inc., Diamond Bar, Irvine, CA, USA). Right and left ventricular ultrahigh-density mapping was conducted in all patients using a 3D electroanatomic mapping system (CARTO PRIME Mapping module, Biosense Webster, Irvine, CA, USA) aiming >1000 mapping points. Bipolar voltage mapping of the left ventricle was conducted during sinus rhythm, intrinsic atrial rhythm or ventricular pacing by ante- and retrograde access with a multielectrode catheter (PENTARAY, Biosense Webster). Epicardial access was assessed by dry epicardial puncture with conventional needles if the clinical VA was morphologically suspected to be of epicardial origin, or in case of ineffective endocardial ablation attempts. A steerable sheath (Agilis Epi, St. Jude Medical, Saint Paul, MN, USA) was inserted into the epicardial space. Low-voltage areas were defined as bipolar voltage of <1.5 mV in endocardial mapping and 5–8 mV in epicardial mapping. Programmed ventricular stimulation was performed before ablation in all patients for tachycardia induction. Catheter ablation targeted induced VA as well as substrate modification of abnormal intracardiac EGMs based on high-density mapping. Local abnormal ventricular activity (LAVA) was defined according to previously published criteria [15]. If VA was inducible and hemodynamically tolerated, then local activation time (LAT) mapping and a novel vector-based mapping algorithm (CARTO Coherent module, Biosense Webster) were implemented to analyze arrhythmia propagation. In case of focal tachycardia patterns, the location of earliest activation was targeted for ablation. In cases with underlying re-entrant mechanisms, zones of slow conduction based on coherent mapping were ablated. Pace mapping of induced and clinical VA was performed using an automated VA-template-matching algorithm (CARTO PASO module, Biosense Webster). Radiofrequency ablation was delivered in a power-controlled mode with a maximum of 40 W, a maximum temperature of 43 °C and a low rate of 30 mL/min using an open, irrigated-tip ablation catheter (ThermoCool, Biosense Webster, Inc., Diamond Bar, Irvine, CA, USA). The primary procedural endpoint was the non-inducibility of any sustained VA at the end of the procedure, defined as complete success. Non-inducibility of the clinical VA was defined as partial success. Elimination of all LAVA was defined as the procedural endpoint in terms of substrate modification. The ablation workflow is depicted in Figure 1.

### 2.4. Coherent Mapping

Coherent mapping is a novel feature of the CARTO 3 system providing an additional method to visualize electrical arrhythmia propagation by color coding and direction vectors [13]. The coherent map is derived automatically from all points that are taken during activation mapping of the tachycardia as well as sinus rhythm. Areas with probable conduction barriers are identified by the module if activity recording is failing, if the bipolar voltage is below 0.03 mV or if conduction velocity vectors are missing. Identified areas represent zones of slow conduction or blocks and are displayed in brown color. The arrhythmia propagation is then visualized by implementation of brown color and projected dynamic conduction velocity vectors. Slow conduction is highlighted by thicker conduction velocity vectors.

### 2.5. Postprocedural Care and Follow Up

All patients were transferred to a wake-up area after the procedure. Extubating was performed postprocedural while the patient was still in the electrophysiology laboratory before being transferred to a wake-up area and the ward. Patients who underwent epicardial puncture for ablation were monitored for at least 24 h post-procedure in the intensive care unit. Epicardial drainage by a pigtail catheter was continued after epicardial ablation until no relevant amount of pericardial effusion could be aspirated and serial echocardiographic imaging showed no evidence of pericardial effusion. The regularly scheduled in-hospital stay after ablation was at least two days. Clinical FU was conducted at our outpatient clinic after 3, 6 and 12 months, including assessment of the clinical history and 12-lead ECG. In patients without intracardiac devices, 72 h Holter ECGs were performed. Subsequently, clinical visits were conducted every 12 months at our outpatient clinic. Device interrogation was conducted in patients with implantable cardiac devices at presentation. If available, device interrogation by remote monitoring was conducted every 4 weeks or immediately in case of sustained arrhythmia episodes. Arrhythmia recurrence was defined as any documented sustained VA with or without ICD therapy. Inadequate ICD therapy due to atrial arrhythmia or sensing issues was not counted as VT recurrence after ablation.

### 2.6. Statistical Analysis

Continuous data were summarized as means +/− standard deviations and categorical data as absolute and relative frequencies (*n*, %). Arrhythmia-free survival was estimated with the use of Kaplan–Meier curves. All *p*-values were two-sided and a *p*-value < 0.05 was considered statistically significant. All calculations were performed with statistical analysis software (IBM^®^ SPSS^®^, Armonk, NY, USA).

## 3. Results

### 3.1. Baseline Characteristics

A total number of 74 patients were analyzed (Table 1). Sixty-five patients (87.8%) were male and mean the age was 65 ± 11.44 years. Mean body mass index was 28.57 ± 6.71 kg/m^2^. All patients suffered from structural heart disease. ICM was the underlying disease in 35 patients (47.3%) and NICM was the underlying disease in 39 patients (52.7%). Mean left ventricular ejection fraction was 33.8 ± 9.9%. Average glomerular filtration rate was 61 ± 15.4 mL/min. Oral anticoagulation was present in 46 patients (62.2%), with 50% of the patients receiving vitamin K antagonists and 50% receiving DOAC. Antiplatelet therapy was present in 26 patients (35.1%). Continuous antiarrhythmic drug therapy before catheter ablation was administered in 48 patients (64.9%). Of these patients, 91.7% were on continuous medication with amiodarone and 8.3% were on continuous medication with sotalol.

### 3.2. Procedural Data

Mean procedure duration was 167 ± 27.68 min with a mean fluoroscopy time of 11 ± 16.5 min with a mean area dosage product of 1319.04 ± 121.67 cGy*cm^2^. Epicardial puncture and ablation was performed in 10 patients (13.5%). Mean ablation time was 34 ± 16.21 min. Mean mapping time during sinus rhythm was 7 ± 5.18 min for the right ventricle and 26 ± 9.88 min for the left ventricle. Mean number of points taken during left ventricular voltage mapping was 2044.76 ± 1269.81 points. Mean time of tachycardia mapping was 9 ± 1.78 min. Average number of points taken for local activation times (LAT) during tachycardia was 1286.62 ± 217.29 points. Mean volume was 132.89 ± 43.11 mL for the right ventricular and 281.62 ± 101.75 mL for the left ventricle. The average extent of low-voltage areas was 19.6 ± 17.18% (Table 2).

**Figure 2:**
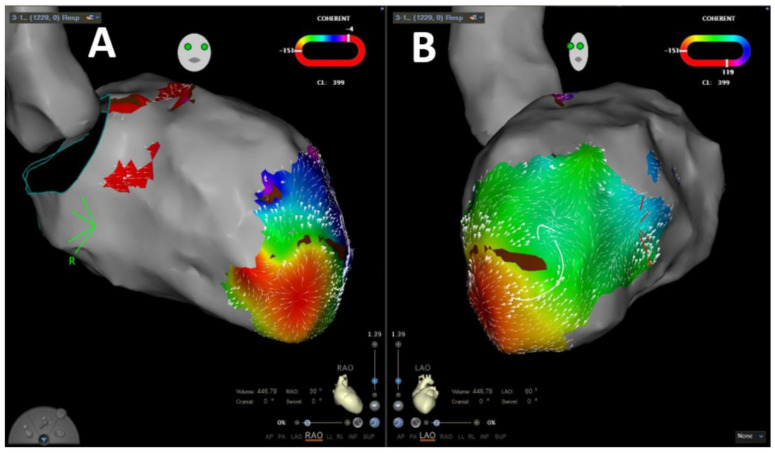
Representative image of velocity-vector-based coherent mapping for ventricular arrhythmia propagation mapping in RAO (**A**) and LA (**B**) projections.

**Figure 3:**
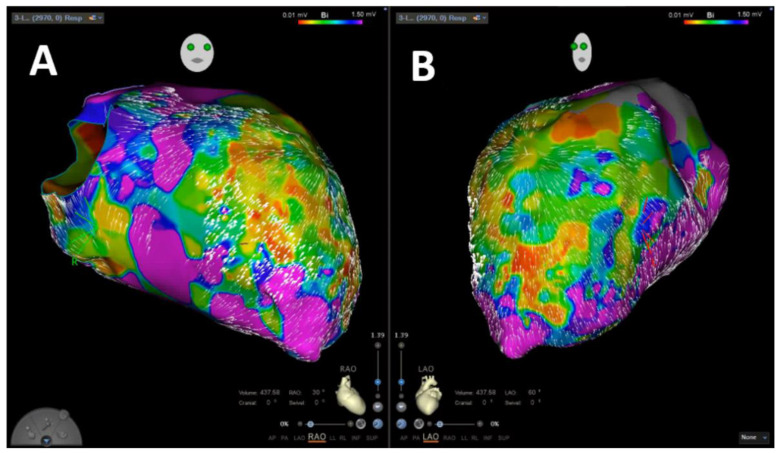
Representative image of left ventricular velocity-vector-based coherent mapping in combination to bipolar voltage mapping in RAO (**A**) and LAO (**B**) projections.

VA was inducible in 65 patients (87.8%). In 31 patients (41.9%), the induced VA was stable, and mapping could be performed. In 29 patients (93.5%), stable VA during the procedure termination by ablation, based on findings during coherent mapping, was achieved. Figure 2 shows a representative example of coherent mapping in a left ventricular VA which terminated under coherent-guided ablation. Successful pace mapping of induced VA and ablation was performed in 27 patients (36.5%). Extensive substrate modification was conducted in 66 patients (89.2%) with successful complete elimination of all detectable LAVA. Figure 3 shows a representative example of a combined bipolar voltage and coherent mapping to guide tachycardia-specific ablation as well as substrate modification in a patient with a large left ventricular low-voltage area. The primary ablation endpoint of non-inducibility of any VA at the end of the procedure was met in 70 patients (94.6%). In 4 patients (5.4%), partial success with non-inducibility of the clinical VA at the end of the procedure, but with remaining inducibility of other sustained VA, was achieved. Major complications occurred in 3 patients (4.1%): 1 patient (1.4%) experienced pericardial effusion without cardiac tamponade and no need for intervention, and 2 patients (2.7%) were diagnosed with false aneurysm at the puncture site without the need for surgical intervention. No major complications occurred in patients who underwent epicardial puncture.

### 3.3. Follow Up

Mean FU duration was 221.64 ± 25.78 days. Recurrence of sustained VT after ablation was observed in 15 patients (20.3%). Mean time to recurrence was 247 ± 183.51 days. ICD therapy after ablation occurred in 12 patients (16.7%) after ablation with anti-tachycardia pacing (ATP) in 11 patients (15.3%) and ICD shock delivery in 7 patients (9.7%). During the follow-up period 9 patients died (12.2%), with 7 patients (77.8%) experiencing a non-cardiovascular event. Of the remaining two patients (22.2%), one died due to cardiogenic shock after recurrent VA and the other one died due to low-output syndrome in the context of chronic left ventricular dysfunction (Table 3).

### 3.4. Arrhythmia-Free Survival

**Figure 4:**
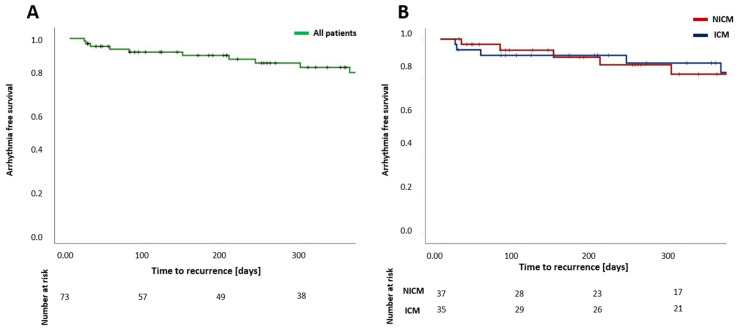
Estimation of arrhythmia-free survival after catheter ablation of ventricular arrhythmia for all patients (**A**) and for patients with ICM in comparison to patients with NICM (**B**) after a single procedure. NICM—nonischemic cardiomyopathy; ICM—ischemic cardiomyopathy.

Survival analysis using the Kaplan–Meier method showed an arrhythmia-free survival of 85.1 ± 4.7% after 12 months for all patients (Figure 4A). In patients with ICM, arrhythmia-free survival was 87.6 ± 5.9% after 12 months, and in patients with NICM, arrhythmia-free survival was 81.9 ± 7.6% after 12 months (Figure 4B). A significant difference between arrhythmia recurrence in ICM or NICM was not observed (*p* = 0.737)

### 3.5. Ventricular Arrhythmia Burden

**Figure 5:**
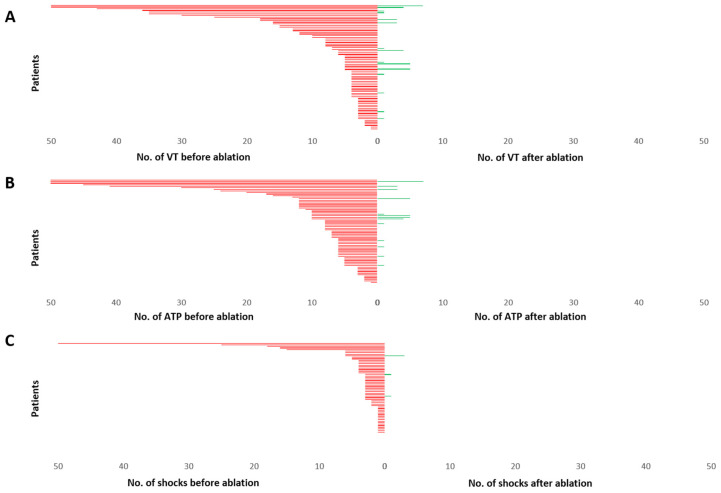
Burden of VA (**A**), ATP (**B**) and ICD shock delivery (**C**), displayed for each patient during 6 months before ablation and 6 months after ablation. Each patient is represented by a single line arranged from highest number of episodes to lowest. The upper values for events have been cut at 50. A significant reduction in mean number of VA, ATP and ICD shocks could be observed. VT—ventricular tachycardia; ATP—anti-tachycardia pacing; No.—number.

Mean number of VA six months before ablation was 10.23 ± 1.69 episodes. Mean number of ATP and ICD shocks before ablation was 11.75 ± 2.01 and 4.22 ± 1.23, respectively. After ablation mean number of VA observed during follow up was 0.53 ± 0.16 episodes, mean number of ATP was 0.55 ± 0.17 and mean number of ICD shocks was 0.07 ± 0.05. VA (*p* < 0.001), ATP (*p* < 0.001) and ICD shock burden (*p* = 0.001) were significantly reduced in all patients after VA ablation (Figure 5).

## 4. Discussion

The present study elucidates the effectivity of VA ablation in patients with SHD based on high-density mapping, implementing a novel velocity-vector-based mapping algorithm. To the best of our knowledge, this is the first study systematically analyzing a coherent-based mapping strategy for ablation of VA.

The main findings of the study are as follows:

1. Ablation of VA based on high-density, velocity-vector-based mapping was performed with short procedural duration and low complication rates.
2. Intraprocedural termination of induced VA was achieved in a high number of patients using the novel mapping algorithm.
3. Ablation of VA based on the present approach was associated with high acute and mid-term success rates in patients with ICM, as well as in patients with NICM.
4. A significant reduction in VA burden and consecutively of ICD therapy burden was observed in all patients after VA ablation.

### 4.1. Velocity Vector Mapping for Guidance of VA Ablation

Today, catheter ablation of VA is an established therapy in patients with recurrent VA refractory to medical treatment or in patients who poorly tolerate medical therapy [16]. Since the 1990s, radiofrequency-based catheter ablation of VA using irrigated catheters has been evolving [17,18,19]. In the current era, VA ablation is mainly based on activation, entrainment, pace and substrate mapping, implementing point-by-point mapping and multielectrode mapping. Defining reliable endpoints of VA ablation is an ongoing challenge. Until today, non-inducibility of VA to programmed stimulation is mostly seen as the gold standard in terms of procedural endpoints. Observational data concerning procedural strategies such as substrate modification especially in patients presenting with unmappable VA show promising results but have not undergone prospective comparison to approaches aiming at tachycardia mapping yet [20].

Coherent mapping is a novel feature of the CARTO 3 system providing an additional method to visualize electrical arrhythmia propagation based on velocity vector identification, enabling identification of critical arrhythmia components. Recently, this novel mapping module has been evaluated in ablation of complex atrial tachycardia and was shown to be effective in identification of tachycardia mechanisms [13,21]. To the best of our knowledge, this mapping module has not been systematically evaluated for VA ablation before. In general, ventricular 3D geometry is complex, and identification of tachycardia mechanisms is difficult, resulting in a need for elaborated mapping strategies. In the present study, this novel mapping algorithm was implemented in the procedural workflow. We found that inducible VA could be terminated at ablation, guided by coherent mapping in over 90% of cases. Our results show for the first time that coherent mapping is feasible for guiding ablation of scar-related VA; therefore, it is a promising tool for VA ablation that should be further evaluated in larger trials aiming at refinement of parameters for ventricular velocity vector mapping.

### 4.2. Effectiveness of VA Ablation Implementing Current Mapping Technologies

In the present study, we observed high acute and mid-term procedural success rates. Acute procedural success defined as non-inducibility of any VA at the end of the procedure was achieved in over 90% of the patients. Survival estimation based on clinical follow up showed an arrhythmia-free survival after 12 months of 85% in the overall patient population, with high effectiveness even in patients with NICM. According to the randomized DANISH trial, primary prevention implantation of ICD did not show mortality benefits in patients with NICM [22]. However, the value of VA ablation as a tool of secondary prevention in NICM on patient mortality has to be further evaluated. Comparing our results to past study results, a relevant increase in arrhythmia-free survival can be observed. In the SMS trial, survival rates of approximately 65% were observed 12 months post-ablation [11]. The VANISH trial observed similar arrhythmia-free survival rates after 12 months with approximately 60% of patients without VA recurrence [12]. In the CALYPSO trial, VT recurrence was observed in 62% of the patients with a median time to recurrence of 75 days [23]. Investigators of the BERLIN-VT study found a mean arrhythmia-free survival 12 months after VA ablation of approximately 60% [24]. In the VTACH trial, an arrhythmia-free survival of approximately 60% after 12 months was observed [10]. The relatively great difference between previously published randomized data and our success rates is based on several relevant differences. First, the above-mentioned studies were conducted between 2007 and 2017, where ablation approaches were based on point-by-point mapping, without the possibility of multielectrode mapping. Due to technological developments and greater global operator experience today, significantly lower success rates in the past seem plausible. Importantly, none of the above-mentioned studies had uniform procedural endpoints, and in some cases, conventional ablation was performed based on fluoroscopy only without additional mapping [11]. Our ablation approach implemented high-density mapping in all patients to guide a combination of substrate modification and tachycardia mapping in a patient-tailored approach. Furthermore, in our study, we performed epicardial puncture and ablation consequently in patients with epicardial substrate, potentially improving procedural success. However, the patient number and follow-up duration in our study might account in part for the observed difference also. Additionally, comparison of patient cohorts from different studies suffers from its typical limitations. Nevertheless, our workflow of VA ablation was highly effective and with a low frequency of procedure-related complications resulting in significantly reduced VA burden in treated patients. Coherent mapping might represent a novel technological tool in the context of VA ablation, potentially leading to lower arrhythmia recurrence rates.

### 4.3. Limitations

The study had an observational design and therefore has the typical limitations in terms of data acquisition. Furthermore, the study population was relatively small. Cardiac magnetic resonance imaging was not performed prior to ablation and might have added further information on VA foci. A potential limitation might be that the present study represents feasibility and outcome of VA ablation in a highly experienced electrophysiological center with trained staff, so that our findings may differ in less-experienced centers. The most important limitation of the study is the lack of a control group, so that a definite conclusion on the possible facilitation of VA ablation by combination of coherent mapping and high-density LAT cannot be made. Further studies in the field are warranted.

## 5. Conclusions

Catheter ablation of VA in patients with structural heart disease is safe and associated with high success rates in terms of reducing VA burden and, consecutively, the delivery of ICD therapy. High-density mapping in combination with coherent mapping possibly facilitates the understanding of tachycardia mechanisms, providing targets for effective ablation.

## Figures and Tables

**Table 1:** Baseline data.

Male	65 (87.8)
Female	9 (12.2)
Age [years]	65 ± 11.44
BMI [kg/m²]	28.57 ± 6.71
GFR [mL/min]	61 ± 15.4
NICM	39 (52.7)
ICM	35 (47.3)
LV-EF [%]	33.82 ± 9.88
ICD	72 (97.3)
LVAD	5 (6.8)
Antiarrhythmic medication	48 (64.9)
Amiodaron	44 (91.7)
Sotalol	4 (8.3)
Oral anticoagulation	46 (62.2)
DOAC	23 (0.5)
Vitamin K antagonist	23 (0.5)
Antiplatelet medication	26 (35.1)
VT storm	15 (20.3)
Epicardial access	10 (13.5)

BMI—body mass index; GFR—glomerular filtration rate; NICM—nonischemic cardiomyopathy; ICM—ischemic cardiomyopathy; LV-EF—left ventricular ejection fraction; ICD—implantable cardioverter defibrillator; LVAD—left ventricular assist device; DOAC—direct oral anticoagulation; VT—ventricular tachycardia.

**Table 2:** Procedural data.

Procedure time [min]	167 ± 27.68
Fluoroscopy time [min]	11 ± 5
Fluoroscopy dosage [yGym²]	1319.04 ± 121.67
Mapping time RV in sinus rhythm [min]	7 ± 5.18
Mapping time LV in sinus rhythm [min]	26 ± 9.88
Points voltage mapping LV [n], %	2044.76 ± 1269.81
Mapping time tachycardia [min]	9 ± 1.78
Points LAT in tachycardia [n], %	1286.62 ± 217.29
RV volume [mL]	132.89 ± 43.11
LV volume [mL]	281.62 ± 101.75
Low voltage area [%]	19.6 ± 14.18
Ablation time [min]	34 ± 16.21
VT inducible [n], %	65 (87.3)
VT mapping possible [n], %	31 (41.9)
Termination of VT by ablation [n], %	29 (93.5)
Pace-mapping performed [n], %	27 (36.5)
Substrate modification performed [n], %	66 (89.2)
Non-inducibility of any VT at end of procedure [n], %	70 (94.6)

RV—right ventricle; LV—left ventricle; LAT—local activation time; VT—ventricular tachycardia.

**Table 3:** Follow-up data.

Duration [days]	221.64 ± 25.78
VT recurrence [n], %	15 (20.3)
Mean time to recurrence [days]	247 ± 183.51
ICD therapy after ablation [n], %	12 (16.7)
ATP [n], %	11 (15.3)
ICD shock [n], %	7 (9.7)
Death during FU [n], %	9 (12.2)
Death due to non-cardiogenic cause [n], %	7 (77.8)
Death due to cardiogenic cause [n], %	2 (22.2)

VT—ventricular tachycardia; ICD—implantable cardioverter defibrillator; ATP—anti-tachycardia pacing; FU—follow up.

## Data Availability

The data underlying this article will be shared on reasonable request to the corresponding author.
